# Hyperoxia increases arterial oxygen pressure during exercise in type 2 diabetes patients: a feasibility study

**DOI:** 10.1186/s40001-015-0194-5

**Published:** 2016-01-08

**Authors:** Robert Rozenberg, Robert T. Mankowski, Luc J. C. van Loon, Janneke G. Langendonk, Eric J. G. Sijbrands, Anton H. van den Meiracker, Henk J. Stam, Stephan F. E. Praet

**Affiliations:** Subdivision MOVEFIT-Sports Medicine, Department of Rehabilitation Medicine, Erasmus University Medical Center, Wytemaweg 80, 3000 CA Rotterdam, The Netherlands; Department of Human Movement Sciences, NUTRIM School for Nutrition, Toxicology and Metabolism, Maastricht University Medical Centre, Universiteitssingel 50, 6229 ER Maastricht, The Netherlands; Section of Pharmacology, Vascular and Metabolic Diseases, Department of Internal Medicine, Erasmus University Medical Center, ‘s-Gravendijkwal 230, 3015 CE Rotterdam, The Netherlands

**Keywords:** Diabetes type 2, Hyperoxia, Exercise, Oxygen, Dose

## Abstract

**Objective:**

The study investigated the feasibility and potential outcome measures during acute hyperoxia in type 2 diabetes patients (DM2).

**Methods:**

Eleven DM2 patients (7 men and 4 women) were included in the study. The patients cycled (30 min at 20 % W_max_) whilst breathing three different supplemental oxygen flows (SOF, 5, 10, 15 L min^−1^). During hyperoxic exercise, arterial blood gases and intra-arterial blood pressure measurements were obtained.

**Results:**

Arterial pO_2_ levels increased significantly (ANOVA, *p* < 0.05) with SOF: 13.9 ± 1.2 (0 L min^−1^); 18.5 ± 1.5 (5 L min^−1^); 21.7 ± 1.7 (10 L min^−1^); 24.0 ± 2.3 (15 L min^−1^). Heart rate (HR) and pH increased significantly after terminating administration of hyperoxic air.

**Conclusions:**

An SOF of 15 L min^−1^ appears to be more effective than 5 or 10 L min^−1^. Moreover, HR, blood pressure, blood lactate and pH are not recommended as primary outcome measures.

## Background

Breathing a hyperoxic gas mixture has been shown to acutely enhance power output (W) by 8–13 % [1–6], increase oxygen uptake ($\dot{V}{\text{O}}_{ 2}$) by 10–14 % [2, 3, 6–10], decrease blood lactate level [11] and lower perceived exertion [7] during aerobic type of exercise. Both healthy subjects and COPD patients show improved exercise performance with hyperoxia [12–14]. These findings suggest that certain other clinical populations with impaired cardiovascular and/or pulmonary fitness levels might benefit from exercise under hyperoxic conditions as well. Patients with type 2 diabetes (DM2) might be good candidates for hyperoxic exercise training as previous research indicated that DM2 patients frequently have a reduced diffusion capacity of the lungs (8–25 %), inversely related to blood glucose levels as well as duration and severity of DM2 [15–17]. Pathophysiological mechanisms explaining the impaired pulmonary function may be micro-angiopathy, chronic inflammation and autonomic neuropathy [16, 18] resulting in a diminished alveolar micro-vascular reserve [15, 17, 19–21]. Impaired alveolar gas exchange in DM2 patients has been shown to correlate with a lower $\dot{V}{\text{O}}_{ 2}$ and workload capacity during aerobic type of exercise [22].

Although beneficial effects of exercise under hyperoxic conditions have been reported for different types of chronic disease populations [1, 4, 8, 23–26], experimental data on an effective dose of hyperoxic air during exercise in DM2 patients are still lacking. Despite the ongoing debate on oxygen transport and consumption [50], increased oxygen availability in arterial blood may improve intracellular transport and uptake of active muscle tissue, and subsequently improve exercise performance. In accordance, the aim of the present feasibility study was to establish an effective dose of supplemental oxygen in DM2 population as a basis to guide and optimise future hyperoxic exercise training protocols.

## Methods

### Subjects

Eleven patients diagnosed with DM2 for at least 2 years and not taking anti-hypertensive medication were screened and included at the outpatient clinic at Erasmus University Medical Center in Rotterdam, the Netherlands. The characteristics of the eligible patients are presented in Table 1. Out of 22 screened patients, a total of six patients were not willing to participate in the hyperoxic exercise intervention following the maximal exercise test. Four patients were excluded from the hyperoxic experiment because it was not possible to introduce an intra-arterial catheter in the radial artery. One patient was excluded from our study because of abnormally high lactate levels during exercise and was diagnosed with mitochondrial encephalomyopathy, lactic acidosis, and stroke-like episodes (MELAS) syndrome afterwards. Baseline characteristics of excluded patients were not different from the experimental group. Included subjects gave their informed consent to participate in the study, approved by the medical ethical committee of the Erasmus University Medical Center in Rotterdam (ISRCTN number: NTR2299).

**Table 1:** Subject characteristics

(n = 11)	Mean ± SD
Sex (M:F)	7:4
Age (years)	56.3 ± 6.3
T2D duration (years)	10.5 ± 6.6
Weight (kg)	87.7 ± 16.5
Length (cm)	171.1 ± 11.0
BMI (kg/m^2^)	30.1 ± 6.1
Abdominal circumference (cm)	100 ± 13
Fat percentage^a^ (%)	33.9 ± 9.1
Fasting glucose (mmol/L)	11.3 ± 3.0
HbA1c (%)	8.3 ± 1.3

No significant differences (p < 0.05)

^a^Based on skinfold measurements (Durnin and Womersley 1969)

### Procedures

**Fig. 1:**
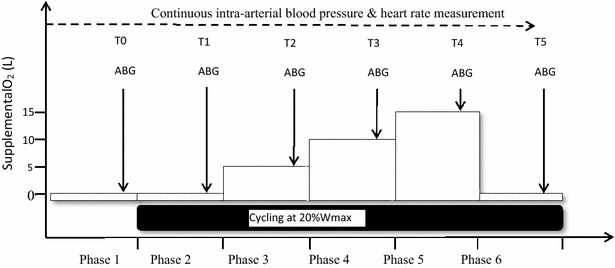
Hyperoxic exercise protocol. *ABG* arterial blood gas, *RPE* rate of perceived exertion (Borg score)

Prior to the hyperoxic exercise session all subjects performed a maximal exercise test on a cycle ergometer (Jaeger ER800) using an incremental workload (1.85 W/6 s for men, 1.2 W/6 s for women). The oxygen uptake ($\dot{V}{\text{O}}_{ 2}$) (Oxycon Pro, Viasys, Houten, Netherlands) and heart rate (HR) (Polar wear-link, Finland) were measured continuously. A second visit was scheduled within 1–3 weeks following a maximal exercise test. During the second visit subjects performed a hyperoxic exercise session. The bout consisted of 25 min of submaximal cycling at 20 % of the maximal workload capacity (*W*_max_). The workload was chosen to ensure that the subjects reach steady state, based on the assumption that the anaerobic threshold is at least 40 % *W*_max_ in DM2 patients. After calibration patients underwent the Allen’s test, and subsequently beat-to-beat blood pressure was obtained through a percutaneous intra-articular catheter in the radial artery of the non-dominant hand [51]. Data were registered in a computer and analysed using specialised software (Beat scope, Finapres Medical Systems, Amsterdam, the Netherlands). It was performed to minimise the risk of ischaemia of the hand. The exercise protocol consisted of 6 phases: 5 min of rest without supplemental oxygen flow (SOF), 10 min without SOF, 5 min with 5 L min^−1^ SOF, 5 min with 10 L min^−1^ SOF, 5 min with 15 L min^−1^ SOF, 5 min without SOF (Fig. 1). A stage duration of 5 min was chosen to reach steady state during, at least, the last 2 min of each stage [27, 28]. The last stage was added to assess the effect of cessation of SOF.

The SOF was administered directly into a face mask (without a reservoir bag, Teleflex Inc. Hudson RCI adult Multi-Vent air entrainment mask), allowing the inhalation of room air to meet the subjects’ ventilatory demands. In our study, we chose to dose oxygen as a fixed flow quantity, instead of a fixed inspirational fraction with a maximum of 15 L min^−1^. This design was chosen to match the possibilities of the standard facilities for supplemental oxygen available in most primary and secondary healthcare settings. During the last min of each phase arterial blood gas and the rate of perceived exertion (Borg score) [29] were obtained.

### Statistical analysis

An independent sample *T* test was used to analyse the baseline characteristics and maximal exercise test. We used a single-factor ANOVA with repeated measures to compare the means of the given variables during different hyperoxic exercise phases. Differences with a *p* value <0.05 were considered significant. The Bonferroni adjustment was applied. Data were presented as mean ± SD.

## Results

### Participants

The characteristics of the included participants are presented in Table 1.

### Maximal exercise test

The average maximal oxygen uptake (*V*O_2max_) of the subjects was 1.83 ± 0.59 L min^−1^. The mean* V*O_2max_ was on average ~24 % (*p* < 0.05) below the average of a healthy population of the same age, weight, length and sex based on the regression equations by Fairbarn et al. [30]. Maximal HR was not significantly different form the predicted values according to the Tanaka regression equation [31]. The results of the maximal exercise test are presented in Table 2.

**Table 2:** Maximal exercise test

	Maximal	Predicted	% Predicted	AT	Ratio AT/max
Load (W)	145.5 ± 61.9	182.1 ± 74.3^a^	84 ± 24*	85.1 ± 38.5	0.58 ± 0.12*
Load/weight (W/kg)	1.70 ± 0.71	2.52 ± 0.82	69 ± 23*	0.99 ± 0.41	0.58 ± 0.12*
$\dot{V}{\text{O}}_{ 2}$ (ml/min)	1830 ± 593	2499 ± 773^a^	76 ± 21*	1334 ± 354	0.75 ± 0.13*
$\dot{V}{\text{O}}_{ 2}$ (ml/min kg)	21.4 ± 7.0	28.9 ± 8.8	76 ± 24*	15.5 ± 4.0	0.75 ± 0.13*
HR (bpm)	155 ± 18	169 ± 5^b^	92 ± 10	128 ± 17	0.83 ± 0.08*
RER	1.09 ± 0.09			0.92 ± 0.07	0.85 ± 0.07*
Systolic blood pressure (mmHg)	180 ± 30				
Diastolic blood pressure (mmHg)	79 ± 12				
RPE (Borg	15.8 ± 2.8				

*AT* anaerobic threshold using V-slope method

^a^Using the Fairbarn and Wasserman equations (Fairbarn et al. [30])

^b^Using the Tanaka equation (Tanaka et al. [31])

* Significant difference (*p* < 0.05)

### Hyperoxic exercise: blood gas analysis

**Fig. 2:**
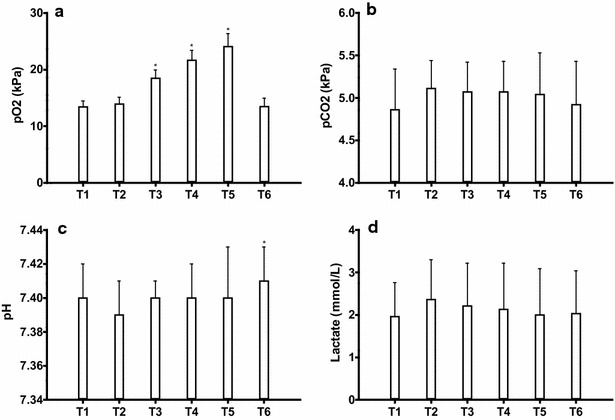
Blood gas analysis **a** pO_2_ levels during hyperoxic exercise. *T3–5 are significantly different from T1, T2 and T6, and each other (p < 0.05). **b** pCO2 levels during hyperoxic exercise. No significant changes (p < 0.05). **c** pH levels during hyperoxic exercise. *T6 is significantly higher than T2 and T3 (p < 0.05). **d** Lactate levels during hyperoxic exercise. No significant changes (p < 0.05)

All included subjects were able to complete 25 min of submaximal hyperoxic exercise. Arterial pO_2_ levels (Fig. 2a) did not change immediately after starting the exercise (T1–2). The arterial pO_2_ increased significantly with increased SOF (T2–T5) in a dose-dependent manner and returned to baseline after cessation of the SOF administration (T5–T6). The arterial pCO_2_ (Fig. 2b) did not change significantly in response to exercise under different SOF.

There was a decreasing trend of the pH (Fig. 2c) at the onset of the exercise bout (T1–T2), while pH increased significantly (*p* value) during the last step when the supplemental oxygen flow administration was stopped (T5–T6).

### Hyperoxic exercise: HR, blood pressure and rate of perceived exertion

**Fig. 3:**
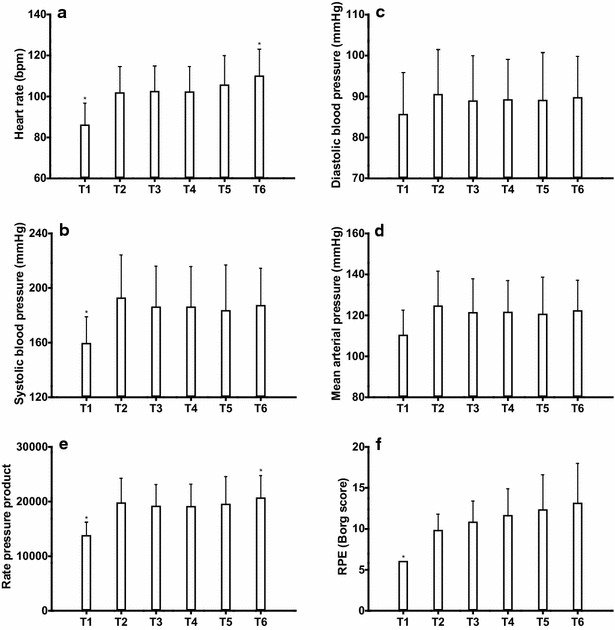
Cardiovascular response and rate of perceived exertion **a** HR during hyperoxic exercise. *T1 is significantly lower than T2–6. T6 is significantly higher than T1, T3, and T4 (p < 0.05). **b** Systolic blood pressure during hyperoxic exercise. *T1 is significantly lower than T2, T3 and T6 (p < 0.05). **c** Diastolic blood pressure during hyperoxic exercise. No significant changes (p < 0.05). **d** Mean arterial blood pressure during hyperoxic exercise. No significant changes (p < 0.05). **e** Rate pressure product during hyperoxic exercise. *T1 is significantly lower than T2–6. T6 is significantly higher than T1 and T3 (p < 0.05). **f** Rate of perceived exertion (Borg score) during hyperoxic exercise. *T1 is significantly lower T2–T6 (p < 0.05)

The HR (Fig. 3a) increased significantly after the onset of the exercise bout (T1–T2), and remained unchanged during the SOF phases (T2–T5) and increased after stopping the SOF (T5–T6). After an initial increase of systolic and diastolic blood pressure after starting the exercise (T1–2), systolic, diastolic and mean arterial blood pressure (Fig. 3b–d) did not change during the SOF. Furthermore, the rate pressure product (Fig. 3e) showed a significant increase after stopping the SOF (T5–T6). The rate of perceived exertion (Fig. 3f) increased with the start of exercise (T1–2) and did not increase significantly during exercise (T2–T6).

## Discussion

We tested the feasibility of hyperoxic exercise and dose–response in type 2 diabetes patients. The main finding of this feasibility study was that exercise under SOF 15 L min^−1^ increased pO_2_ more effectively than lower doses (5 and 10 L min^−1^) in DM2 patients (Table 3).

**Table 3:** Hyperoxic exercise session

	T1	T2	T3	T4	T5	T6
pO2 (kPa)	13.4 ± 1.1	13.9 ± 1.2	18.5 ± 1.5^a^	21.7 ± 1,7^a^	24.0 ± 2.3^a^	13.5 ± 1.5
Oxygen content (mmol/L)	8.3 ± 1.0	8.3 ± 0.9	8.3 ± 1.0	8.6 ± 1.2	8.4 ± 1.0	8.2 ± 1.1
pCO2 (kPa)	4.9 ± 0.5	5.1 ± 0.3	5.1 ± 0.4	5.1 ± 0.4	5.0 ± 0.5	4.9 ± 0.5
pH	7.40 ± 0.02	7.39 ± 0.02	7.40 ± 0.01	7.40 ± 0.02	7.40 ± 0.03	7.41 ± 0.02^b^
Lactate (mmol/L)	1.96 ± 0.80	2.36 ± 0.94	2.21 ± 1.01	2.13 ± 1.09	2.00 ± 1.09	2.03 ± 1.10
Heart rate (bpm)	86 ± 11^c^	102 ± 13	102 ± 13	102 ± 12	106 ± 12	110 ± 13^c^
Systolic blood pressure (mmHg)	159.4 ± 19.5^d^	192.6 ± 31.7	186.1 ± 30.0	186.0 ± 29.7	183.3 ± 33.5	187.1 ± 27.3
Diastolic blood pressure (mmHg)	85.6 ± 10.2	90.5 ± 11.0	88.9 ± 11.1	89.1 ± 9.9	89.0 ± 11.7	89.7 ± 10.1
Rate pressure product	13,626 ± 2483^e^	19,722 ± 4569	19,106 ± 4000	19,062 ± 4115	19,471 ± 5112	20,627 ± 4137^e^
Mean arterial pressure (mmHg)	110.2 ± 12.4	124.5 ± 17.1	121.3 ± 16.6	121.4 ± 15.6	120.5 ± 18.2	122.2 ± 15.0
Rate of perceived exertion (/20)	6.0 ± 0.0^f^	9.8 ± 2.0	10.8 ± 2.6	11.6 ± 3.3	12.3 ± 4.3	13.1 ± 4.9

^a^T3–5 are significantly different from T1, T2 and T6, and each other (p < 0.05)

^b^T6 is significantly higher than T2 and T3 (p < 0.05)

^c^T1 is significantly lower than T2–6. T6 is significantly higher than T1, T3, and T4 (p < 0.05)

^d^T1 is significantly lower than T2, T3 and T6 (p < 0.05)

^e^T1 is significantly lower than T2–6. T6 is significantly higher than T1 and T3 (p < 0.05)

^f^T1 is significantly lower T2–T6 (p < 0.05)

### Technical feasibility

From a technical perspective, our results demonstrate that supplemental oxygen, applied with a standard open facemask (5–15 L min^−1^), results in significant increases in arterial pO_2_ levels during exercise. Higher pO_2_ at increased SOF (i.e. 5, 10 and 15 L min^−1^) suggests a dose-dependent effect. The pO_2_ levels obtained from the radial artery during hyperoxic exercise in the present study (24.0 ± 2.3 kPa) were comparable with the arterial pO_2_ levels measured by Plet et al. in healthy subjects. Administration of 55 % of oxygen improved maximal oxygen uptake by 12 % during cycle-ergometry in comparison with normoxic exercise [9]. Other studies investigating the influence of hyperoxia during exercise found slightly higher pO_2_ levels of approximately 40 kPa obtained from the femoral artery with an inspired oxygen fraction of 60 % [32–34]. Taken together, our data show that supplemental oxygen applied during submaximal exercise via a standard open face mask increases arterial pO_2_ levels. Additional oxygen availability could compensate for the diminished diffusion capacity, endothelial function and low aerobic capacity seen in most DM2 patients [15, 17, 19–21]. The latter suggests that a hyperoxic training study in DM2 patients could be a potential solution in a medical fitness centre, since no special equipment is needed other than an open facemask and standard gas cylinders with O_2_. However, before investigating training effects under hyperoxic conditions, this warrants further controlled trials on cardiovascular and pulmonary function in DM2 patients. The results will improve our understanding on whether additional oxygen during exercise may improve oxidative metabolism in populations such as DM2 with deficient cardiovascular and respiratory function.

### Patient recruitment and study population

Despite the invasive nature of our study and the fact that use of antihypertensive medication was an exclusion criterion for the present study, the majority (=76 %) of the eligible DM2 patients that were approached in our outpatient clinic were willing to participate in our feasibility study. Although, a training study requires a more long-term commitment, the willingness to participate in our feasibility study indicates that it might be possible to recruit a sufficient and representative proportion of subjects for a randomized clinical trial on the medium-term effects of hyperoxic exercise training.

In accordance with previous studies [35–38], the mean VO_2max_ of the investigated patient sample is well below the average of the healthy population, even when corrected for a high BMI. High HbA1c and fasting glucose levels showed that our overweight subjects had poorly regulated DM2. As such, the present study population may not be representative for the general well-controlled DM2 population. Long-term adherence has been reported to vary substantially (10–80 %) in conventional exercise programs for DM2 patients [39–44]. However, effects of hyperoxic exercise training in other patient populations, with a reduced alveolar and capillary diffusion capacity [12–14], showed the anticipated increase in exercise capacity. Less perceived exercise intensity and improvement of exercise performance will also motivate overweight and with poorly regulated DM2 patients to adhere to hyperoxic exercise training.

### Potential outcome measures

In contrast with previous hyperoxic exercise studies [8, 9, 33, 45–47], we observed no change in HR, blood pressure or rate of perceived exertion during exercise while increasing the supplemental oxygen flow during exercise. However, after stopping administration of SOF we observed a significant increase in HR and rate pressure product [HR * systolic blood pressure (SBP)]. The cardiovascular response during phase 6 indicates that hyperoxia lowers the cardiovascular burden during submaximal steady-state exercise in patients with DM2. A number of physiological mechanisms might explain why, in comparison with previous hyperoxic exercise studies, SOF did not lower HR and systemic blood pressure during phases 3–5 in our experimental setup. First, it is possible that even at an exercise intensity of 20 % $\dot{V}{\text{O}}_{{ 2 {\text{ max}}}}$ max, our patients were not completely in a steady-state condition during phases 3–5. Second, in comparison with previous hyperoxic exercise studies, the absolute exercise intensity may have been too low to cause a significant drop in HR, blood pressure or the rate of perceived exertion (Borg score). Third, the arterial wall stiffening in combination with the diabetes-related endothelial dysfunction may have impaired a normal vascular response to hyperoxia [48, 49].

### Limitations of the study

Unfortunately, for medical ethical reasons (invasive study) it was difficult to add a healthy control group or different oxygen conditions. Because of this limitation, we can only speculate about the physiological reason for this abnormal response to hyperoxic exercise. Arterial blood gas collection (arterial blood withdrawal) was vastly limited because of impaired structure of arterial walls in the DM2 patients. These invasive methods may be replaced by non-invasive study measures in the future studies such as bio-impedance cardiography and near-infrared spectroscopy. Nevertheless, the present feasibility study suggests that HR, blood pressure and rate of perceived exertion may not be suitable primary outcome measures for a hyperoxic training study in unfit DM2 patients. Instead, direct assessment of the VO_2max_ should be considered in a hyperoxic training study to monitor and document change in exercise performance.

## Conclusions

Based on arterial pO_2_ measurements, a supplemental oxygen flow of 15 L min^−1^ appears sufficient to compensate for impaired alveolar and capillary oxygen transport and/or consumption in DM2 patients. Based on this feasibility study, we propose to first investigate acute effects of various inspiratory oxygen fractions on the cardio-respiratory system and speed of oxygen uptake kinetics. This will improve our understanding on potential exercise performance enhancement benefits of supplementary oxygen. This would warrant future studies to investigate the medium- and long-term benefits of hyperoxic exercise training in patients with DM2.
